# Effects of voice on emotional arousal

**DOI:** 10.3389/fpsyg.2013.00675

**Published:** 2013-10-01

**Authors:** Psyche Loui, Justin P. Bachorik, H. Charles Li, Gottfried Schlaug

**Affiliations:** ^1^Department of Neurology, Beth Israel Deaconess Medical Center and Harvard Medical School, BostonMA, USA; ^2^Department of Psychology, Wesleyan University, MiddletownCT, USA

**Keywords:** emotion, music, arousal, perception, gender, aging

## Abstract

Music is a powerful medium capable of eliciting a broad range of emotions. Although the relationship between language and music is well documented, relatively little is known about the effects of lyrics and the voice on the emotional processing of music and on listeners' preferences. In the present study, we investigated the effects of vocals in music on participants' perceived valence and arousal in songs. Participants (*N* = 50) made valence and arousal ratings for familiar songs that were presented with and without the voice. We observed robust effects of vocal content on perceived arousal. Furthermore, we found that the effect of the voice on enhancing arousal ratings is independent of familiarity of the song and differs across genders and age: females were more influenced by vocals than males; furthermore these gender effects were enhanced among older adults. Results highlight the effects of gender and aging in emotion perception and are discussed in terms of the social roles of music.

## Introduction

The ability to detect emotion in speech and music is an important task in our daily lives. The power of the human voice to communicate emotion is well documented in verbal speech (Fairbanks and Pronovost, 1938; Scherer, 1995) as well as in non-verbal vocal sounds (Skinner, 1935), and the human voice is thought to convey emotional valence, arousal, and intensity (Laukka et al., 2005) via its modification of spectral and temporal signals (Fairbanks and Pronovost, 1938; Bachorowski and Owren, 1995). The use of the human voice to convey emotion is abundant and vital developmentally as in the case of infant-directed speech (Trainor et al., 2000), and can be accurately identified by people of different cultures (Bryant and Barrett, 2008), suggesting that emotion communication may be a universal function of the human voice. Furthermore, the inability to detect emotional signals in voices is associated with psychopathy (Bagley et al., 2009), thus highlighting the importance of emotional identification in the auditory modality in every human functioning.

Music is another form of sound communication that conveys emotional information. To understand the perception of emotions in music, one model that has been validated by psychological and physiological studies is as a two-dimensional space that treats affect as two separable dimensions of valence and arousal (Russell, 1980). This valence-arousal model is well validated with musical stimuli (Balkwill and Thompson, 1999; Bigand et al., 2005; Ilie and Thompson, 2006; Steinbeis et al., 2006; Grewe et al., 2007). Studies investigating why and how music is able to influence its listeners' moods and emotions (Sloboda, 1991; Terwogt and van Grinsven, 1991; Balkwill and Thompson, 1999; Panksepp and Bernatzky, 2002; Gosselin et al., 2007) have identified ratings for musical stimuli that drive changes in each of these two factors independently. Arousal is a measure of perceived energy level, ranging from low (calming) to high (exciting) (Krumhansl, 1997; Gosselin et al., 2007; Sammler et al., 2007). Orthogonally, valence is the polarity of perceived emotions, and ranges from negative (sad) to positive (happy) (Krumhansl, 1997; Schubert, 1999; Dalla Bella et al., 2001). Multidimensional scaling (MDS) studies have verified that valence and arousal are separable measures, that may be independently manipulated in experimental conditions (Bigand et al., 2005; Vines et al., 2005).

Given that music and the voice may both be strong modulators of emotions, vocal music could be a medium with emotional power. Several studies have investigated the cognition and perception of vocal lyrics in songs. Serafine et al. (1982) studied the effect of lyrics on participants' memory for songs. Results showed that melody recognition was near chance unless the melody's original words (i.e., words that were presented with the music during encoding) were present, suggesting that music and speech were combined into a single coherent object when encoded in the same stream. More recently, Weiss et al. (2012) examined the effect of timbre (including voice) on memory and preference for music. Results showed that melodies with the voice were better recognized than all other instrumental melodies. The authors suggest that the biological significance of the human voice provides a greater depth of processing and enhanced memory.

Few studies have investigated the combination of music and speech in emotion perception. In an investigation of the effects of varying stimulus parameters in music and speech on perceived emotion, Ilie and Thompson (2006) showed that emotional ratings for music and speech concurred in most emotion ratings, except that manipulations of pitch height resulted in different directions of valence change for music and speech. Interaction effects between music and speech were again observed, suggesting that the combination of speech with music may result in complex and non-additive effects on emotion.

As music and speech are both auditory stimuli that vary over time, a fundamental question regarding emotion perception of these auditory sources concerns the time-course of emotional responses. Approaches that have been used to investigate the time-course of emotion perception in music include online responses made during the presentation of music, and offline responses made after hearing musical excerpts. Using both offline techniques of categorization and MDS (Perrot and Gjerdingen, 1999; Bigand et al., 2005), subjective emotional ratings performed after hearing short musical stimuli showed that a musical segment as short as 250 ms in duration is sufficient to elicit a reliable emotional response. However, these emotional ratings were influenced by the *post-hoc* cognitive appraisal of emotional content within music after their presentation, as well as the emotional experience elicited by music during its presentation. Using continuous emotional ratings in the two-dimensional space of valence and arousal maximizes the influence of emotion perceived online during the presentation of musical stimuli (Schubert, 2004). In previous work using the two-dimensional continuous paradigm (Bachorik et al., 2009), participants took an average of 8.3 s to initiate movement signifying an emotional judgment.

The present study adopts both continuous (online) and discrete (offline) subjective ratings to investigate effects of vocals on perception of arousal in music. In addition to exploring the effects of vocals on arousal in music in a temporally sensitive manner, further questions arise concerning the factors that moderate participants' emotional response to the presence of vocals in songs. As previous studies have shown that age and gender may contribute to personality characteristics, which in turn influence musical preference (Rentfrow and Gosling, 2003), we examined the interaction of arousal ratings with age and gender, while controlling for effects of familiarity on arousal ratings. Subjects were presented with excerpts from two versions of well-known songs, one with vocals and one without (with all other variables in the songs being the same), and made continuous as well as discrete ratings of perceived arousal, as well as familiarity ratings, for each version of each song.

## Materials and methods

### Participants

Fifty participants (25 females and 25 males) were recruited from the greater Boston metropolitan area via advertisements in daily newspapers. Participants ranged from 19 to 83 years of age (median = 37), and were representative of the Boston metropolitan area in their ethnic distribution. All participants reported having no neurological and/or psychiatric disorders and had normal IQ as assessed by Shipley abstract scale scores (Shipley, 1940). Written informed consent, approved by the Institutional Review Board of the Beth Israel Deaconess Medical Center, was obtained from all participants. Each participant was reimbursed at an hourly rate for participating.

### Stimuli

The stimuli consisted of 32 unique musical excerpts, each 60 s long. Vocal and instrumental versions of 16 songs were chosen from commercially available songs (see Table 1 for a list of all songs used). All excerpts were normalized for loudness and each excerpt was briefly faded in (0.5 s) at the beginning of the stimulus and out (0.5 s) at the end. The stimuli were divided into two blocks of 16 trials each; each block consisted of both versions (vocal/instrumental and instrumental only) of 8 songs. Excerpts ranged in tempo between 49 and 177 beats per minute.

**Table 1:** **Excerpts of song stimuli**.

**Artist**	**Title**	**One-min selection**
Whitney Houston	I will always love you	1:30–2:30
Bette Midler	Wind beneath my wings	0:30–1:30
Donna Summer	Last dance	0:30–1:30
Bryan Adams	Everything I do	0:30–1:30
Sonny and Cher	I got you babe	0:30–1:30
The Carpenters	Close to you	0:30–1:30
Simple minds	Don't you forget about me	0:30–1:30
Madonna	Like a virgin	0:30–1:30
Lionel Richie and Diana Ross	Endless love	0:30–1:30
Barbara Streisand	The way we were	0:30–1:30
Mr. Mister	Broken wings	0:30–1:30
Alanis Morsette	You oughta know	0:30–1:30
The Police	Every breath you take	0:30–1:30
Gloria Gaynor	I will survive	0:30–1:30
R.E.M.	Losing my religion	0:30–1:30
The Beatles	Can't buy me love	0:30–1:30

Experiments were conducted using an Apple Powerbook G4 with a 15.4″ LCD screen using custom-made stimulus presentation software (Sourcetone, LLC). Audio was presented via Altec Lansing AHP-712 headphones, and participants used a mouse and a Flightstick Pro USB joystick to input their responses to the stimuli.

### Procedure

Over the course of two separate testing sessions, each participant completed two trial blocks. Order of trial block presentation was counterbalanced between subjects. Each of the 16 excerpts in each trial block was played in a randomized order, and for each stimulus presentation, the participant's task was the same: to use the joystick to respond, in real time, to the levels of emotional valence (defined as positive or negative emotion induced by the music) and arousal (defined as a stimulating or calming feeling induced by the music) of the music via an onscreen cursor in a two-dimensional grid. The joystick controlled the motion of the cursor in a 640 × 640 resolution grid, and data about the position of the joystick and the position of the cursor was sampled with a frequency of 10 Hz. Centering the joystick caused the cursor to stop moving but did not center the cursor in the grid onscreen.

After the end of each musical excerpt, subjects had additional tasks to rate the degree of valence and arousal perceived in each excerpt (on a scale of 0–4, where 4 is highest, 2 is neutral, and 0 is lowest). Participants also provided subjective ratings of familiarity (on a scale of 0–4, with 0 being “never heard” and 4 being “actively listen to; personally own song”) after rating the degree of emotional arousal and valence.

### Data analysis

Continuous ratings for valence and arousal (X and Y axes on the two-dimensional rating space, respectively) were digitized and exported for each trial of each subject from the stimulus presentation program and analyzed using in-house software. Pairwise *t*-tests were conducted for each time point comparing subjects' valence and arousal ratings for vocal and instrumental versions of each song. A false-discovery rate *post-hoc* adjustment was used to minimize Type I error.

Discrete valence and arousal ratings were used as the dependent variable in a mixed design ANOVA with between-subject factors of age (two levels: old vs. young, with a median split at the age of 37) and gender (male vs. female) and the within-subject factor of song vocals (instrumental vs. vocals). Paired *t*-tests were run comparing music with and without vocals in familiarity, liking, chills, and intense emotional responses.

## Results

Continuous arousal ratings revealed that the vocal versions were more arousing overall. The average continuous ratings were higher in the vocal version than in the instrumental version in 15 out of 16 songs. This was confirmed using a pairwise *t*-test at every point in the time-series comparing arousal ratings in vocal and instrumental conditions indicating significant difference at the FDR-corrected alpha level of 0.05 in at least one time point between vocal and instrumental versions in 12 out of 16 songs. Among these 12 songs, 11 showed a significant arousal-enhancing effect of vocals, whereas only one song showed the opposite effect. In contrast to arousal ratings, continuous valence ratings only showed significantly higher valence ratings at the *p* < 0.05 (corrected) level for at least one point in 4 out of 16 songs, and significantly lower valence ratings for at least one point in two songs.

**Figure 1:**
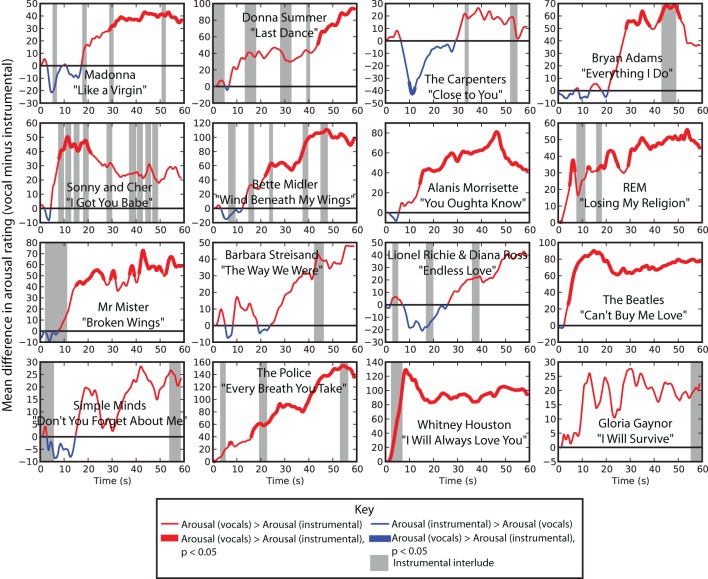
**Difference between vocal and instrumental versions of each song over time.** Positive difference means that arousal ratings for vocal pieces were higher than for their instrumental counterparts; negative differences means that arousal ratings were higher for instrumental pieces than for vocals. Thick lines indicate a significant difference at the 0.05 (FDR corrected) alpha level.

Figure 1 shows the difference between average arousal rating between vocal and instrumental versions as functions of time for each of the 16 songs. Red line segments indicate a higher arousal rating in vocal versions compared to instrumental versions whereas blue line segments indicate the opposite effect. Bold lines indicate significant differences at the *p* < 0.05 (FDR-corrected) level and gray bars behind the graph indicate instrumental interludes within the vocal versions of each song.

Online ratings indicated that, as shown in Figure 1, the arousal-enhancing effect of vocals was more pronounced later within each piece. The trend toward higher arousal ratings in the vocal versions began at an average of 10 s after the onset of each song, however, this was variable depending on the song (SEM = 2.6 s). The presence of instrumental interludes within each song was uncorrelated with the difference in arousal ratings. Songs that contained non-verbal vocal portions (Whitney Houston, Barbara Streisand, and Mr. Mister songs in the sample) showed a similar effect size as songs containing verbal vocals, suggesting that the presence of the human voice, rather than recognizable words, led to the increase in arousal.

**Figure 2:**
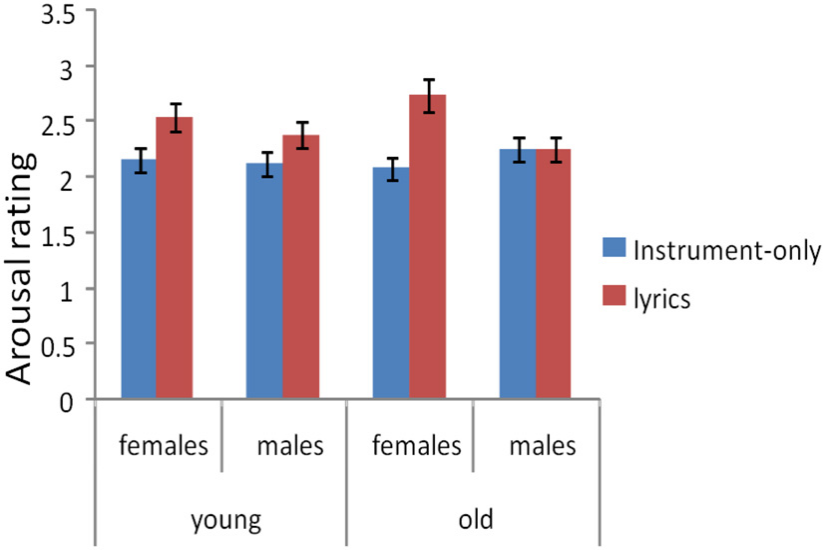
**Three-way interaction between vocals, sex, and age on arousal ratings**.

The effect of vocals on arousal was confirmed in discrete as well as continuous arousal ratings. Using the discrete arousal rating as the dependent variable, the mean arousal rating for instrumental versions of the musical excerpts was 2.25 (SEM = 0.07) whereas the mean arousal rating for vocal versions was 2.60 (SEM = 0.06). A highly significant main effect of vocals on arousal was observed, *F*_(1, 96)_ = 1389.5, *p* < 0.001, indicating that songs with vocals were rated as more highly arousing than their instrument-only counterparts (Figure 2). Participants also reported liking the vocal versions more than the instrumental version, with a mean of 2.75 vs. 2.48, respectively [*t*_(49)_ = −3.486, *p* < 0.001]. The same effect was not observed in discrete valence ratings [*F*_(1, 96)_ = 1.17, n.s.].

Using discrete arousal ratings as the dependent variable, we next attempted to tease apart the groups of participants who were or were not susceptible to the effects of vocals on arousal by assessing the demographics (gender and age) of each participant and comparing the mean difference between vocals and instrumental versions across demographic groups. A significant main effect of gender was observed for all arousal ratings, with ratings by females being higher [*F*_(1, 96)_ = 4.186, *p* = 0.04]. Furthermore, a significant interaction between vocals and gender was observed on arousal ratings: *F*_(1, 96)_ = 11.9, *p* = 0.001, confirming that the positive effect of vocals on arousal ratings was stronger for females than for males (Figure 2). Although no significant main effect of age was present [*F*_(1, 96)_ = 0.013, n.s.], a significant three-way interaction was observed on arousal ratings between gender and age [*F*_(1, 96)_ = 4.17, *p* = 0.04], with older females being more emotionally influenced by vocals than younger females, but older males being less influenced by vocals than younger males (Figure 2).

Familiarity ratings revealed that participants found songs with vocals to be significantly more familiar than the instrumental version [mean ratings: vocals = 2.63, instrumental = 1.872; *t*_(49)_ = −9.319, *p* < 0.001]. To investigate the effects of vocals on arousal while controlling for the effect of familiarity, a one-way ANCOVA was conducted on the dependent variable of discrete arousal rating with the factor of vocals (instrumentals vs. vocals), with the covariate of familiarity rating (0 through 4). Results showed a significant effect of vocals [*F*_(1, 97)_ = 4.2, *p* = 0.043] even with a significant effect of familiarity [F_(1, 97)_ = 6.3, *p* = 0.014], suggesting that the contribution of vocal stimuli to arousal was significant even after controlling for an increase in familiarity for vocal pieces.

## Discussion

Our results indicate that the presence of vocals generally enhances participants' arousal ratings, and were not limited to the effects of familiarity but were moderated by the gender and age of the participant. Vocal sounds and music engage multiple common resources in the brain, resulting in interactions between music and speech as assessed by tasks that tap into perception, cognition and emotion (Serafine et al., 1982; Besson et al., 1998; Ilie and Thompson, 2006). However, little research has investigated the time-course of the impact that vocals may have on arousal perception in music. Using a naturalistic and ecologically valid setting of popular songs with and without vocal content, the present study attempted to address the specific question concerning the relationship between vocals and perceived arousal in music. While the present study uses ecologically valid stimuli and identifies arousal differences attributable to the use of vocals within music, future research may be done to tease apart specific components of the vocals (e.g., words, timbre, sung melody) that most affect perceived arousal.

Based on continuous (online) and discrete (offline) subjective ratings of valence and arousal for identical musical excerpts with and without vocal content, we observed that the presence of vocals generally increases ratings of arousal but not of valence. The emotionally enhancing effect of vocals on arousal is shown in both online (continuous) and offline (discrete) ratings of subjective arousal, and is not limited to verbal lyrics but appears to generalize to non-verbal songs containing the human voice. Online ratings revealed that participants required an average of 10 s (SEM = 2.6 s) of music before differentiating vocal versions from instrumental versions; this was congruent with previous reports using a similar continuous ratings paradigm (Bachorik et al., 2009) showing that participants required an average of 8.3 s to initiate emotional ratings when listening in real time. Furthermore, the enhancing effect of vocals is not limited to familiarity, as shown by an ANCOVA revealing that effects of vocals were significant even after statistically controlling for the contribution of familiarity ratings.

It is interesting to speculate on why valence is less affected by vocals compared to arousal. One possibility is that vocals affected valence both positively and negatively depending on the listener and depending on the song, resulting in increased variability. Another possibility is that valence is already much determined from other structural features of music such as modality (major vs. minor keys) and melodic contour, leaving little changes that the added vocals could bestow upon the perceived valence of each song. The relative impact of structural features of a piece on its perceived valence vs. arousal may be an avenue for future studies.

As music with vocals has additional components of timbre, melody, and words, the present experiment design could be followed up by assessing the effect of an additional lead instrument on arousal ratings in a non-vocal control condition. However, the selection of the most appropriate additional lead instrument in such a design is non-trivial, as only a highly systematic match in timbre between the voice and the chosen test instrument would provide a true test of the possible confound of voice timbre. Future experiments should seek to identify a timbral match of the voices used in these naturalistic song stimuli in order to define a timbre-matched control condition. Nevertheless, in the current analysis we identify song sections that do not include words as a possible means to de-confound the relationship between voice and lyrics, and as the increase in arousal ratings is observed even for sections of the songs that include non-verbal vocals, the results suggest that the use of vocals, rather than of lyrics within the music, may be driving the increase in arousal.

When offline ratings were compared by the demographic variables of gender and age, results revealed the types of participants who were most sensitive to the arousal-enhancing effect of vocals. Females were more inclined to report perceiving higher arousal in vocal songs compared to males. These effects are exaggerated among older participants. One possible explanation for the gender effect is that the need to detect emotional signals rapidly may be more evolutionarily advantageous for women. Supporting evidence along this possible evolutionary basis of gender-bias in selecting for emotion in vocal content comes from electrophysiological literature showing that the dishabituation of emotional voice content is more robust in females, and is furthermore regulated by estrogen levels (Schirmer et al., 2008). Regarding the three-way interaction between the effect of vocals with gender and with age, one possibility is that the song stimuli—popular songs ranging from the 1960s to the 1990s—chosen for this experiment are more familiar to older individuals than to younger ones. However, the fact that the effect of vocals on arousal was still significant after controlling for the contribution of familiarity suggests that the influence of vocals on arousal was above and beyond the influence of familiarity. Another possible explanation stems from how individuals of different ages identify with music, with possible sociological effects of changing standards of gender equality throughout the decades that may help explain the observed gender by age interaction. As young adults rely on musical preferences to communicate and understand each other's personality profiles (Rentfrow and Gosling, 2006), it would seem that younger individuals, especially females and individuals who rely on external feedback and social pressures for self-perception, may be more easily aroused by music that is representative of their own culture and the personality profile they wish to convey. Since most popular music is written with vocals, it stands to reason that younger listeners looking to identify themselves with popular taste would find music more arousing when presented with vocals. As the emotional content of songs is highly influenced by our identity as captured by demographic variables such as age and gender, future work should seek to refine our understanding of emotion perception in music and language by placing it in broader sociological and biological contexts.

The present results from continuous and discrete ratings, obtained during and after music listening, support the central notion that the combination of vocal and instrumental sounds in music could produce a more pronounced effect on emotional arousal, but not on valence, compared to instrumental music alone. The arousal-enhancing effect of vocals increases over the duration of most songs and is moderated by demographic factors such as age and gender. Results have implications for our understanding of the emotion and meaning of music, and will bear relevance for ongoing efforts to model and predict the emotional content of music (Nagel et al., 2007) for therapeutic as well as commercial applications.

### Conflict of interest statement

The authors declare that the research was conducted in the absence of any commercial or financial relationships that could be construed as a potential conflict of interest.
